# News and Notes

**Published:** 1911-12

**Authors:** 


### NEWS AND NOTES

### PROGRAM FOR THE ELEVENTH SEMI-ANNUAL SESSION OF THE CHATTAHOOCHEE VALLEY MEDICAL AND SURGICAL ASSOCIATION.

To Be Held At LaGrange, Ga., Tuesday and Wednesday, Jan. 16-17, 1912. Place of Meeting, Elks Home.

FIRST DAY.

Call to Order 12 M.

Invocation—by Rev. H. D. Phillips, LaGrange, Ga.

Address of Welcome, behalf City of LaGrange—Hon. E. R. Bradfield, LaGrange, Ga.

Address of Welcome, behalf Troup County Medical Society —F. M. Ridley, Sr., M. D., LaGrange, Ga.

Response in behalf of the C. V. M. and S. A.—T. Jos. Dean, M. D., Union Springs, Ala.

Annual Address of the President.

AFTERNOON SESSION—2 P. M.

Report of Two Cases of Tumor in Jaw—W. L. Cooke, M. D., Columbus, Ga. Leaders in discussion: J. A. Goggans, J. L. Bowman.

The Right Iliac Region and the General Practitioner—by H. S. Bruce, M. D., Opelika, Ala. Leaders in discussion: C. S. Yarbrough, W. T. Langley, J. L. Campbell.

The Management of Typhoid Convalescence—B. F. Rea. M. D., LaFayette, Ala. Leaders in discussion: B. H. Brock. J. H. McDuffie.

A Plea for the Child with a Running Ear—H. M. Lokey,

M. D., Atlanta, Ga. Leaders in discussion: T. E. Mitchell, H. S. Persons.

Occular Headaches—Paul S. Mertens, M. D., Montgomery, Ala. Leaders in discussion: Geo. W. Chambers, W. G. Harrison and Geo. H. Cooper.

Gynecology in General Practice—A. L. Harlan, M. D., Alex. City, Ala. Leaders in discussion: R. S. Hill, F. M. Ridley, Jr.

The Significance of Ascites—W. P. Dickinson, M. D. LaFayette, Ala. Leaders in discussion: H. T. Hamner, Velpeau Langley.

Traumatic Neuroses—L. M. Gaines, M. D., Atlanta, Ga. Leaders in discussion: H. P. Cole, B. L. Wyman.

Experience of a Tuberculosis Specialist—J. M. Anderson, M. D., Pinedale, Ga. Leaders in discussion: L. M. Gaines, E. C. Thrash.

Treatment of Acute Lobar Pneumonia—G. A. Pate, M. D., Five Points, Ala. Leaders in discussion: Geo S. Murray, W. W. Stevenson.

Operative Treatment of Recent Fractures—F. K. Boland, M. D., Atlanta, Ga. Leaders in discussion: H. B. Disharoon, Gaston Torrance, J. L. Campbell.

Glaucoma—Geo. H. Cooper, M. D., Opelika, Ala. Leaders in discussion: H. S. Persons, W. G. Harrison.

NIGHT SESSION.

Unprotected Public Dangers—C. A. Cary, D. V. M., Auburn, Ala.

The Contribution and Claim of the Medical Profession—W. G. Harrison, M. D., Birmingham, Ala.

SECOND DAY—MORNING SESSION 8:00 A. M.

Malaria from a General Practitioner's Standpoint—G. H. Moore, M. D., Opelika, Ala. Leaders in discussion: T. H. Haralson, J. S. Horsley, Sr.

When is Malaria Cured—W. T. Hodges, M. D., Riverview, Ala. Leaders in discussion: C. L. Williams, Thos. F. Taylor.

The Relation of Oral Sepsis to Digestive Disorders—Geo. M. Niles, M. D., Atlanta, Ga. Leaders in discussion: J. N. Baker, J. S. McLester.

Treatment of Gastric and Duodenal Ulcers—J. N. Baker, M. D., Montgomery, Ala. Leaders in discussion: Geo. M. Niles, Geo. S. Murray.

The Demand for Emptying the Gravid Uterus—Hugh McCulloh, M. D., West Point, Ga. Leaders in discussion: F. M. Ridley, Sr., A. L. Harlan, J. R. B. Branch.

The Prevention of Post Operative Gynecological Psychoses —H. P. Cole, M. D., Mobile, Ala. Leaders in discussion: J. R. B. Branch, Hansell Crenshaw.

The Technique of Prostatectomy—E. G. Ballenger, M. D., Atlanta, Ga. Leaders in discussion: M. L. Boyd, F. M. Johnston.

The Treatment of Prostatic Obstruction Before and After Operation—Montague L. Boyd, M. D., Atlanta, Ga. Leaders in discussion: L. C. Morris, Jno. D. S. Davis, H. S. Bruce.

Dysmenorrhea of Nasal Origin, With Report of a Case—Thos. F. Taylor, M. D., Tuskegee, Ala. Leaders in discussion: J. R. B. Branch, J. A. Goggans.

The Importance of City and County Boards of Health—H. W. Terrell, M. D., LaGrange, Ga. Leaders in discussion: H. W. Sanders, J. S. Horsley.

Pruritus Ani—Homer Boatwright, M. D., Carrollton, Ga. Leaders in discussion: H. W. Terrell, J. M. Poer.

Anaphylaxis, and its Relation to the Use of Antitoxins—Katherine Collins, M. D., Atlanta, Ga. Leaders in discussion: J. S. McLester, L. M. Gaines.

The Value of Urethral Catheterization—W. F. Shallenberger, M. D., Atlanta, Ga. Leaders in discussion: E. G. Ballenger, H. S. Munroe, Jno. D. S. Davis.

A Case of Localized Tuberculosis of Skin, Treated by Tuberculin—J. M. Poer, M. D., West Point, Ga. Leaders in discussion: Wm. Wade Harper, E. C. Thrash.

AFTERNOON SESSION.

Menstruation, Normal and Abnormal—J. R. B. Branch, M. D., Macon, Ga. Leaders in discussion: E. G. Jones, Thos. F. Taylor.

Syphilis of Nervous System—J. M. Brawner, M. D., Atlanta, Ga. Leaders in discussion: J. L. Bowman, E. G. Ballenger.

The Present Status of Specifics in the Treatment of Tuberculosis— E. C. Thrash, M. D., Atlanta, Ga. Leaders in discussion: J. M. Poer, J. M. Anderson.

Lobar Pneumonia—J. P. Liles, M. D., Roanoke, Ala. Leaders in discussion: D. T. Langley, J. J. Winn.

Environment in Treatment of Nervous Diseases—Hansell Crenshaw, M. D., Atlanta, Ga. Leaders in discussion: J. N. Brawner, C. A. Dexter.

Nose and Throat Reflexes Causing Cough—R. R. Daly, M. D., Atlanta, Ga. Leaders in discussion: H. R. Slack, Paul S. Mertens.

Herpes—W. T. Lanley, M. D., Opelika, Ala. Leaders in discussion: J. M. Welch, Jas. T. Striplin and G. A. Pate.

Etiology, Pathology and Treatment of Marasmus—E. C. Williams, M. D., Notasulga, Ala. Leaders in discussion: W. H. Moon, Thos. Northen.

Old Opinions vs. the New—Onslow Reagan, M. D., Alex City, Ala. Leaders in discussion: J. P. Motley, W. D. Floyd.

Abdominal Type of LaGrippe—Roy Oswald, M. D., Union Springs, Ala. Leaders in discussion, W. D. Gaines, G. A. Pate.

Executive Session.

Election of Officers.

Adjournment.

---

### OFFICERS OF THE CHATTAHOOCHEE VALLEY MEDICAL AND SURGICAL ASSOCIATION.

President, L. W. Johnston, M. D. ____________ Tuskegee, Ala.
First Vice-President, H. M. Lokey, M. D. ______ Atlanta, Ga.
Second Vice-President, Thos. H. Haralson, M. D. _ Cusseta, Ala.
Secretary-Treasurer, W. J. Love, M. D. ______ Opelika, Ala.

BOARD OF COUNCIL.

J. H. McDuffie, M. D. President ____________ Columbus, Ga.
A. L. Harland, M. D. ______________________ Alexander City, Ala.
Geo. M. Niles, M. D. ______________________ Atlanta, Ga.

H. B. Disharoon, M. D. ---------------------- Roanoke, Ala.
Geo. H. Cooper, M. D. ----------------------- Opelika, Ala.

PROGRAM COMMITTEE.

W. J. Love, M. D. (Chairman) --------------- Opelika, Ala.
Geo. M. Niles, M. D. --------------------------- Atlanta, Ga.
P. M. Lightfoot, M. D. ------------------- Cross Keys, Ala.

RECEPTION COMMITTEE.

F. M. Ridley, Jr., M. D. (Chairman) --------- LaGrange, Ga.
H. W. Terrell, M. D. --------------------- LaGrange, Ga.
Hugh McCullough, M. D. ----------------- West Point, Ga.
J. M. Poer, M. D. ------------------------- West Point, Ga.
B. H. Brock, M. D. ------------------------ Hogansville, Ga.

---

### REGULAR MEETING FULTON COUNTY MEDICAL SOCIETY, THURSDAY, DECEMBER 7, 1911.

Reported by R. R. Daly, M. D.

Dr. G. K. Varden read a paper on "Significance of Enlarged Lymph Nodes in Children," which appears elsewhere in the "Journal."

Dr. W. Z. Holliday said that the role infectious diseases play in this affection is most important. Especially is this true in Diphtheria, when antitoxin is not used early enough or in sufficiently large doses. The glands are pretty sure to be enlarged if left unaided in doing eliminative work in these diseases. Malnutrition is a factor in the etiology and indicates one of the most important lines of treatment. As to drugs he finds the iodides beneficial and prefers iodide of sodium to any other salt. Burnham's soluble iodide is effective and easily administered.

Dr. F. G. Hodgson said that we are likely to err in ascribing a syphilitic etiology to the majority of these enlarged glands in children. If they are caused by marasmus or malnutrition, iodides and other anti-syphilitic treatment are contraindicated.

Dr. J. W. Duncan said that he has seen a good many of these children and believes that the most of them show the efforts of nature to work over the conditions of faulty assimilation. While syphilis does occur in conjunction with some of them, it should not be diagnosed off-hand.

Dr. R. R. Daly said that the reference of the essayist to recurring adenoids suggested incomplete operation rather than anything else. If an adenoid is not only curetted but thoroughly rubbed out by the gauze-covered finger at the time of the operation, there is no "recurrence." If small parts of the gland are left, they may grow and cause trouble. Dr. C. C. Stockard agreed with Dr. Daly as to the adenoid.

Dr. L. B. Clarke said that these glands are not, as a rule, syphilitic, and that moreover they do not belong to the domain of surgery unless purulent. They are not dangerous to life but are indications of low vitality. He uses with great success cod liver oil when the stomach will stand it. When iodides are indicated he prefers the iodide of strontium.

Dr. Varden said he had seen "recurrent" cases of adenoids after skillful men had operated; possibly some of the obstructions to healing were post-operative adhesions. In reply to a question he said scrofula was tuberculosis of the skin as he understood it. The "diathesis" means little or nothing.

Dr. L. B. Clarke read a paper on "Growing Pains," which appears elsewhere in the "Journal."

Dr. F. W. McRae said that in his boyhood on the farm he used to be exposed to all sorts of weather conditions in doing the work and that he had growing pains, he can well remember. They were not rheumatic, however, and he has had none of the symptoms of rheumatism since then. He doesn't like to have a good term abused, but we should always bear in mind the possibility of rheumatism when the child suffers muscular pains anywhere.

Dr. E. Bates Block expressed the opinion that growing pains are unquestionably associated with rheumatism. Skin lesions, arthritis, tonsilitis and subcutaneous fibroid nodules are frequently present in conjunction with the main symptom. There are two schools of belief as to the cause. One is that there is a definite diplococcus determining the trouble, and the other is that intestinal putrefaction and intoxication is the deciding element. The treatment in both is practically the same thus far.

Sixty-five per cent. of the chorea cases have rheumatism within 3 years either before or after the attack.

Dr. C. E. Boynton said that rheumatism is more generally connected with many diseases than we have thought, and mentioned cyclic vomiting, sick headaches, tonsilitis and growing pains.

Recurrent bronchitis and asthma often respond to anti-rheumatic treatment.

A child said to have congenital heart murmurs showed vomiting attacks and temperature, she had very large tonsils that interfered with her swallowing and seemed to indicate operation. Under appropriate eliminative treatment she improved so greatly as to show scarcely any heart murmur and obviate all need of tonsillectomy.

Dr. Daly said that in his experience large tonsils in connection with rheumatic conditions were better removed early. They and the accompanying adenoids interfered with aeration of the blood and the nutrition of the body—just the conditions in which rheumatism, whatever that may be, did its work. Rheumatic cases were shortened in their duration by early and prompt removal of tonsils, especially when there was reason to believe they were avenues of infection.

Dr. G. K. Varden said he agreed with the paper in the main. The fascia is often involved. Scurvy from 6 to 14 months is sometimes an associated symptom.

Dr. Archibald Smith said that "growing pains" were good to lay trouble upon when we did not know what else to do. They are often associated with septic tonsils, scurvy and ricketts.

Dr. Clarke closed by saying that Dr. McRae's growing pains occurred under the very conditions inducing rheumatism.

---

### CHANGE IN RULES.

At a meeting held this day the Board of Health of the City of Atlanta passed the following rules in regard to the handling of contagious diseases in the City.

1. That the Board of Health shall only placard houses for Scarlet Fever and Diphtheria.

2. That all placards shall be reduced in size to a card 4 x 6 inches.

3. That Physicians will only be required to report cases of Smallpox, Scarlet Fever, Diphtheria and Typhoid Fever.

4. That Diphtheria placards will be put up on Backteriological examination as heretofore or on clinical diagnosis, but that quarantine of diphtheria cases will be raised, the premises fumigated, and placards taken down either on a negative culture or by the Physician in charge reporting to the Health Department that all clinical signs of the disease have disappeared, the Health Department keeping up the quarantine for twelve days after such report from the Physician in charge.

5. That a rigid enforcement of the foregoing be instituted by the Health Officer and that cases be made for any and all violations of same.

Very truly,

.... J. P. KENNEDY,

Health Officer.

---

### DR. CARLES E. DE M. SAJOUS,

*Supervising Editor of the New York Medical Journal.*

We have the honor to announce that beginning with issue of December 9, 1911, Dr. Charles E. de M. Sajous, of Philadelphia, becomes the Supervising Editor of the New York Medical Journal. While Doctor Sajous will give up his private visiting practice, he will continue his work as a consulting physician, investigator, teacher and author, and thus be in a position to keep in the closest toush with the needs of the medical profession.

Though born under the American flag, Doctor Sajous received his preliminary education in France. He studied medicine in Philadelphia, graduating with honors from the Jefferson Medical College in 1878. He served for two years as resident physician in the Howard Hospital, and in 1881 was appointed professor of anatomy and physiology in the Wagner Institute of Science, lecturer in the Philadelphia School of Anatomy, and clinical assistant in the laryngological department of Jefferson Medical College, succeeding Dr. J. Solis-Cohen, in 1883, as clinical lecturer and chief of that department. In 1891 Dotor Sajous went to Paris, where he devoted six years to original research. Upon his return, he was appointed Dean of the Medico-Chirurgical College. At the recent reorganization of the medical department of Temple University Doctor Sajous accepted the chair of pharmacology and therapeutics, which he still holds.

The immediate outcome of Doctor Sajous's six years of research work in Paris was the publication of two volumes on Internal Secretions and the Principles of Medicine, a work which gave the author high standing as an original investigator.

Doctor Sajous has had a wide editorial experience, having founded in 1888 the Annual of the Universal Medical Sciences, which he conducted with the collaboration of some of the most eminent physicians in America and Europe, until the publication was abandoned in 1893. The Annual had a circulation of over 500,000 volumes and the Cyclopaedia of Practical Medicine, founded by Doctor Sajous in 1898, to succeed the Annual, and intended more particularly for the general practitioner, has attained a circulation of 240,000 volumes, the seventh edition being now in course of preparation.

The value of Doctor Sajous's services to medical science has been recognized in France by his being made a member of the Legion of Honor, while in Belgium he received the order of Leopold and was made a Knight Commander of the Liberator, besides receiving other titles, both governmental and scientific. In America Doctor Sajous has been president and vice-president of many societies and is a fellow of the College of Physicians of Philadelphia, and of the American Philosophical Society. He brings to bear on the eitorial problems of the New York Medical Journal, a brilliant and well informed mind, wide experience, and a thorough knowlegde of the needs of the American physician.

The publishers of the New York Medical Journal feel that they as well as its readers are to be congratulated upon having obtained the services of Doctor Sajous. Comprehensive and well directed plans have been formulated for enhancing the value and interest of the New York Medical Journal, and in carrying out these plans no pains or expense will be spared to give our readers a medical journal of unprecedented authority and interest.

---

### SPECIAL WESTERN NUMBER.

In furthering the plan of producing special issues of the American Journal of Surgery, composed of contributions by surgeons residing within a certain geographical area, yet of international reputation there will be issued in the early part of 1912 a SPECIAL WESTERN NUMBER of this magazine.

SUBJECTS AND THOSE WHO CONTRIBUTE:

The Operation of Gastroenterostomy. By William J. Mayo, Rochester, Minn.

Operative Treatment for Graves Disease. By George W. Crile, Cleveland, O.

Colonic Intoxcation. By J. E. Binney, Kansas City, Mo.

Practical Points in the Surgical Treatment of Exopthalmic Goitre. By A. J. Ochsner, Chicago, Ill.

Treatment of Foreign Bodies in the Esophagus. By E. Fletcher Ingals, Chicago, Ill.

Brain Surgery Technique. By J. Rilus Eastman, Indianapolis, Ind.

Treatment of Abscesses and of the Necrotic Focii, Resulting from the Use of Salvarsan. By A. Ravolgi, Cincinnati, O.

Treatment of Prostatic Obstructions. By E. O. Smith, Cincinnati, Ohio.

Subject not Announced. B. Tuholske, St. Louis, Mo.

Artificial Tendons and Ligaments in the Surgical Treatment of Paralysis. By Nathaniel Allison, St. Louis, Mo.

Uterine Cancer. By John C. Murphy, St. Louis, Mo.

Arthritis Deformans. By Leonard W. Ely, Denver, Colo.

Acute Inflammation and Flexure of the Sigmoid, as a Causative Factor in Epilepsy, with Special Reference to Treatment. By W. H. Axtell Bellingham, Wash.

The character of contributions prepared by these well-known surgeons are of such a nature as to make this number particularly interesting.

